# Referral for kidney transplantation: a consensus statement from the Brazilian Society of Nephrology

**DOI:** 10.1590/2175-8239-JBN-2025-0110en

**Published:** 2025-08-18

**Authors:** Helady Sanders-Pinheiro, Luiz Gustavo Modelli de Andrade, Tainá Veras de Sandes-Freitas, Laila Almeida Viana, Lucio Requiao-Moura, Luiz Roberto de Sousa Ulisses, Pedro Tulio Monteiro de Castro de Abreu Rocha, Gustavo Fernandes Ferreira, Lilian Pires do Carmo, Lauro Vasconcelos, Alvaro Pacheco-Silva, José Andrade Moura-Neto

**Affiliations:** 1 Sociedade Brasileira de Nefrologia, São Paulo, SP, Brazil.; 2 Universidade Federal de Juiz de Fora, Faculdade de Medicina, Departamento de Clínica Médica, Juiz de Fora, MG, Brazil.; 3 Serviço de Transplante Renal do Hospital Universitário da Universidade Federal de Juiz de Fora, Juiz de Fora, MG, Brazil.; 4 Universidade Estadual Paulista, Departamento de Medicina Interna, Botucatu, SP, Brazil.; 5 Universidade Federal do Ceará, Faculdade de Medicina, Departamento de Clínica Médica, Fortaleza, CE, Brazil.; 6 Rede D’Or, Hospital São Luiz Itaim, São Paulo, SP, Brazil.; 7 Fundação Oswaldo Ramos, Hospital do Rim, São Paulo, SP, Brazil.; 8 Universidade Federal de São Paulo, Departamento de Medicina, São Paulo, SP, Brazil.; 9 NEFROCLÍNICAS, Brasília, DF, Brazil.; 10 Rede Américas, Hospital São Lucas Copacabana, Rio de Janeiro, RJ, Brazil.; 11 Universidade Federal do Rio de Janeiro, Hospital Universitário Clementino Fraga Filho, Unidade de Terapia Intensiva, Rio de Janeiro, RJ, Brazil.; 12 Santa Casa de Juiz de Fora, Unidade de Transplante, Juiz de Fora, MG, Brazil.; 13 Universidade Federal de Minas Gerais, Faculdade de Medicina, Departamento de Clínica Médica, Belo Horizonte, MG, Brazil.; 14 Hospital Evangélico de Belo Horizonte, Serviço de Transplante Renal, Belo Horizonte, MG, Brazil.; 15 Universidade Federal do Espírito Santo, Faculdade de Medicina, Departamento de Clínica Médica, Vitória, ES, Brazil.; 16 Escola Bahiana de Medicina e Saúde Pública, Departamento de Clínica Médica, Salvador, BA, Brazil.

**Keywords:** Kidney Transplantation, Chronic Kidney Disease, Referral, Effective Access to Healthcare Services, Consensus, Clinical Decision Making

## Abstract

Brazil ranks among the top five countries performing kidney transplants, operating the largest public transplantation system in the world. Nevertheless, the number of procedures performed represents less than 40% of demand, and there are significant regional disparities, with areas of the country reporting very low transplantation activity. Among the potential causes of this scenario are the low frequency of referrals for transplant evaluation and referrals made under inappropriate clinical conditions. In this document, the Brazilian Society of Nephrology aims to compile information supporting the selection of kidney transplantation as a renal replacement therapy. The document also details contraindications and patient care after listing and prioritization, in line with the national context. Our goal is to provide a document that serves as a national reference and contributes to streamlining the referral process for kidney transplantation, making it more frequent and effective, thereby expanding candidates՚ access to this treatment

## Initial Considerations

Advanced chronic kidney disease (CKD) represents an increasing burden on healthcare to healthcare systems worldwide, including Brazil. In 2023, it was estimated that approximately 157,000 patients were undergoing dialysis therapy in the country^1^, while around 33,000 were on the waiting list for a kidney transplant^2^. Compared to other modalities of renal replacement therapy (RRT), kidney transplantation offers advantages relating to survival, quality of life, and cost-effectiveness^3,4^. However, despite its benefits, access to transplantation in Brazil remains limited by several factors, such as donation rates that fall short of demand, lack of awareness about the importance of donation, the need for prior discussion with family members regarding individual willingness to donate organs^5^, the location and distribution of transplant centers, selection criteria, and inadequate referral of patients.

Disparities in access to kidney transplantation and the barriers that prevent eligible patients from enlisting represent challenges to making the process more efficient, equitable, and fair. In this context, the “Referral for Kidney Transplantation: a Consensus Statement from the Brazilian Society of Nephrology” proposes evidence-based recommendations and expert opinions to guide patient selection, identification of contraindications, prioritization, and referral, so that decisions are grounded in well-defined clinical criteria and aligned with national realities and legislation. Thus, it provides a simplified, accessible, and practical source for clarifying common questions from non-transplant nephrologists - professionals who play a strategic role in initiating the process that ultimately leads to patient access to kidney transplantation.

## Kidney Transplantation as a Renal Replacement Therapy

***Kidney transplantation should be presented as a RRT option, alongside dialysis modalities, to patients with CKD and risk of progression, as part of conservative treatment, starting at stage G4 [glomerular filtration rate (GFR) 29-15 ml/min/1.73m^2^].***

As CKD progresses to its advanced stage (stage 5: GFR <15ml/min/1.73m^2^), kidney transplantation is the RRT that offers the best outcomes^3,4^. For this reason, when feasible, it is considered the treatment of choice^3,4,6^. These superior outcomes are reflected in increased survival^4^, improved quality of life^3,7^, reduced morbidity^3,4^ and lower costs^8^. The benefit of increased survival has been documented for decades^9^ and remains evident in recent studies, which reveal a 55% higher survival rate following kidney transplantation compared to patients remaining on the waiting list - who are generally the healthiest among those undergoing dialysis^3,4^. Higher survival rates are observed even among deceased donors classified as non-optimal or expanded criteria donors^10^ (age >60 years or between 50–59 years, with a history of hypertension, cerebrovascular cause of death, or serum creatinine ≥1.5 mg/dl at the time of donation)^11,12^. An epidemiological change has been observed in patients diagnosed with CKD and, consequently, in kidney transplant candidates, with an increase in the proportion of patients with higher morbidity. Improved survival has also been observed in these groups, including the elderly^13,14^, diabetic patients^15^, those with multiple comorbidities^14^, on long-term dialysis (>10 years)^16^ or sensitized^17^, as well as in lower-volume transplant centers^18^. In addition to increased survival, kidney transplantation is associated with outcomes that are easily perceived and highly valued by patients, such as improved quality of life^3,19^, measured, for example, by greater participation in daily activities^7^ and lower morbidity (e.g., hospitalizations)^3,4^. From a healthcare system perspective, kidney transplantation generally incurs lower costs and is considered cost-effective, especially in more complex cases, such as those involving very marginal donors [Kidney Donor Profile Index (KDPI) >85]^8^. In pediatric patients, kidney transplantation also promotes improved survival^20^, as well as improvements in the growth trajectory^21^, neurocognitive development^22^, and overall quality of life^23^.

Studies that demonstrate the superiority of kidney transplantation outcomes over other RRTs, however, have been primarily based on data from developed countries with high socioeconomic status^6^. Nevertheless, according to longitudinal data from the Brazilian Dialysis Survey (*Censo Brasileiro de Diálise*), annual mortality among dialysis patients in Brazil has ranged from 17.1% to 20.3% over the past eight years^1^, comparable to rates reported in other countries. Conversely, short- and medium-term survival after kidney transplantation - at 1 and 5 years, respectively - is 97% and 93% for grafts from living donors, and 92% and 83% for grafts from deceased donors, according to data from the latest Brazilian Transplant Registry (*Registro Brasileiro de Transplantes*, RBT) of the Brazilian Association of Organ Transplantation (*Associação Brasileira de Transplante de Órgãos*, ABTO)^2^. Survival rates in Brazil are therefore similar to those reported in studies from other countries^24^. Considering data from both surveys and given the lack of comparative studies in the country, we can hypothesize that the benefit of increased survival after kidney transplantation may also be extrapolated to the Brazilian population. In the national context, an analysis of secondary data from the multicenter cross-sectional study ADHERE BRAZIL, which included 1,105 kidney transplant recipients, assessed quality of life using the WHOQOL-BREF instrument. The results showed that Brazilian kidney transplant recipients had excellent quality of life across all four domains evaluated, with particular emphasis on physical and social relationship domains^25^. In accordance with this, the Clinical Guidelines for the Care of Patients with CKD in the Brazilian Unified Health System, issued by the Ministry of Health, recommend that kidney transplantation be offered as a RRT option^26^.

In summary, it can be considered that the choice of kidney transplantation as RRT should be offered to CKD patients with an expected progression to advanced stages^6^.

## Indications for Kidney Transplantation

***In patients with indication, referral should be made as early as possible, according to the clinical condition of the transplant candidate and respecting the patient’s choice. Two aspects should be considered: the timing of referral to a transplant service and the criteria established in Brazilian legislation for registration on the waiting list.***

***Potential candidates for kidney transplantation should be referred to a specialized center when GFR falls below 20 ml/min/1.73m^2^.***

***For waitlisting, the criteria defined in the Brazilian legislation are as follows:***

***I – Patients undergoing any modality of RRT (hemodialysis or peritoneal dialysis); or***

***II – Patients with GFR lower than 10 ml/min/1.73m^2^; or***

***III- Patients under 18 years of age with a GFR lower than 15 mL/min/1.73m^2^; or***

***IV - Patients with diabetes mellitus who have a GFR below 15 ml/min/1.73m^2^.***

***In the case of candidates for living-donor transplants, donor evaluation may be initiated in patients who are already undergoing RRT or who have GFR below 15 ml/min/1.73m^2^.***

Based on the potential benefits of kidney transplantation as RRT, referral should be made early. The Clinical Guidelines for the Care of Patients with Chronic Kidney Disease in the Brazilian Unified Health System recommend that patients be referred from stage G5, i.e., with GFR below 15 ml/min/1.73m^2 6^. However, there is no consensus on the exact timing for referring patients to a specialized kidney transplant service. Ideally, potential kidney transplant candidates should be referred for evaluation at least six to twelve months before the anticipated initiation of dialysis, to facilitate the identification/investigation of living donors and plan for a possible preemptive transplant^6^. The goal is to reduce the risk of early death by enabling timely access to kidney transplantation. Despite the lack of consensus, considering the benefits of preemptive transplantation, this working group recommends referring patients with GFR below 20 ml/min/1.73m^2^.

Preemptive transplantation offers better clinical outcomes following the procedure, improved quality of life, and economic benefits when compared to dialysis. It is associated with increased graft survival and a lower incidence of acute rejection. It also allows patients to be spared the potential risks associated with dialysis therapy, such as catheter-related infection, adverse cardiovascular effects, and intradialytic complications, including hypotension^27,28^. Despite its benefits, preemptive transplantation is not widely performed worldwide. Between 2000 and 2018, less than 10% (9.3%) of kidney transplants in the United States were performed preemptively. Reports from other countries reveal even lower rates: Spain (5%), Uruguay (5.4%), and Indonesia (2.7%)^28^. Brazil does not have accurate records on the number of preemptive transplants performed; however, data from the ADHERE BRAZIL study showed that only 3.3% of the 1,105 patients included underwent preemptive transplantation^29^. Several factors may account for the low number of this transplant modality: organ allocation policies, patient and healthcare team education, and delayed referral to a nephrologist^30^. In Brazil, preemptive kidney transplantation may be performed not only with living donors, but also with deceased donors, in patients with GFR <10 ml/min/1.73m^2^, or < 15 ml/min/1.73m^2^ in the case of patients under 18 years of age and those with diabetes, as previously mentioned^31^.

In the transplant service, after clinical and laboratory evaluation, potential candidates may be registered on the transplant list, according to the criteria defined by current legislation^31^. Patients with diabetes mellitus and children represent special cases, as morbidity and mortality increase significantly as GFR decreases. For this reason, listing is recommended for patients with GFR <15ml/min/1.73m^2^ in these groups ^6,31^. After RRT initiation, patients with shorter dialysis vintage progress with a lower risk of death and graft loss after kidney transplantation^32, 33, 34^. This difference is already observed even with relatively short periods, such as 6 months of dialysis treatment, and increases progressively with each additional year of therapy. The risk of death increases from 18% to 68% among patients who have been on dialysis for 6 months to >5 years, respectively, while the risk of graft loss ranges from 25% to 46% over the same periods^32^. The Clinical Guidelines for the Care of Patients with Chronic Kidney Disease (CKD) in the Brazilian Unified Health System (SUS) recommends a period of up to 90 days after the initiation of dialysis for official notification concerning the decision to undergo kidney transplantation, as a means of expediting and ensuring access to referral. This choice may be changed at any time, either by the patient or as a result of their clinical condition^26,35^.

Simultaneous pancreas-kidney transplantation may be considered for patients with insulin-dependent diabetes mellitus (type I, latent autoimmune diabetes in adults, MODY, or early onset diabetes) combined with CKD on dialysis or with a glomerular filtration rate lower than 20ml/min/1.73m^2^. When combined kidney transplantation with any other organ (liver or heart) is required, graft allocation follows the specific legislation governing the other organ transplant^31^.

## Contraindications: Absolute, Relative, and Temporary

***The following may be considered absolute contraindications, which therefore do not justify referral for kidney transplant evaluation: untreated malignant neoplasms, progressive degenerative neurological disease, severe vasculopathy involving the iliac arteries, poorly controlled psychiatric illness, illicit substance abuse, organ failure with no possibility of treatment or with indication for combined transplantation (heart and liver).***

***Relative or temporary contraindications may include: untreated coronary artery disease; recent immunological graft loss (< 6 months); recent cerebrovascular stroke (CVS), acute myocardial infarction (AMI), or transient ischemic attack (TIA) (< 6 months for CVS/AMI; < 3 months for TIA); extrarenal activity of systemic disease; active peptic ulcer disease; acute infection; full anticoagulation that cannot be suspended or reversed; and dual antiplatelet therapy.***

***For situations classified as temporary or relative, it is recommended to wait for resolution/evolution of the condition before referral.***

***Given the complexity and difficulty of generalization, it is recommended to advise the patient to obtain a second opinion if they are contraindicated for kidney transplantation after evaluation.***

***As the decision is individualized and based on the evaluation of a multidisciplinary team, we suggest referring the patient to the transplant center for further deliberation in case of uncertainty. Alternatively, the service may be contacted for guidance.***

The consideration that a patient is not clinically eligible for evaluation for kidney transplantation is a topic of considerable debate, since the criteria and thresholds for such assessment are not clearly defined^6^. For the purposes of this consensus, we will categorize contraindications for referral into: **1. Absolute**: those in which there is virtually no doubt regarding the absence of benefit, or in which the risk associated with kidney transplantation is exceedingly high; **2. Relative**: the benefit is likely, but the associated risk is also high - though potentially manageable; in these cases, acceptance of the candidate varies among transplant centers; **3. Temporary**: high risk conditions, but likely to be resolved in the short or medium term. Most centers will not accept patients with absolute contraindications as candidates; therefore, it is suggested that they not be referred. In cases classified as temporary or relative, it is advisable to wait for resolution/evolution of the condition before referral^6^.

There are some conditions that make transplantation more challenging, eventually reducing its success rate. These include transplantation in elderly individuals^36^, obese individuals^37^, people living with human immunodeficiency virus (HIV) infection^38^, hepatitis B, hepatitis C^39^, or in the presence of multiple comorbidities^14^. However, when assessing the benefits of kidney transplantation, staying on dialysis should be used as the comparator rather than transplantation performed under optimal conditions. In this regard, the evidence available to date has consistently demonstrated that transplantation benefits most of these patients, who should therefore be referred for pre-transplant evaluation^14,36, 37, 38, 39^. During this evaluation, the transplantation team conducts a comprehensive assessment of the patient and, if deemed eligible, proceeds with guidance on the control or mitigation of conditions that can be managed, such as comorbidities and viral diseases.

As for neoplasms, if the patient has an active cancer with no prospect of treatment, or is undergoing chemotherapy or radiotherapy, they should not be referred for transplantation at this point. However, after treatment has been completed, the indication for transplantation will depend on the type of cancer and the time elapsed since the end of treatment. Given the complexity of this assessment, we recommend referring the patient to a transplant center, where a multidisciplinary team will evaluate the risk-benefit ratio on an individual basis^6^.

## Referral Flow

***Patients who meet the clinical criteria for transplantation should be referred to accredited centers, following local recommendations, where they will undergo evaluation for subsequent inclusion on the list.***

**Figure 1:**
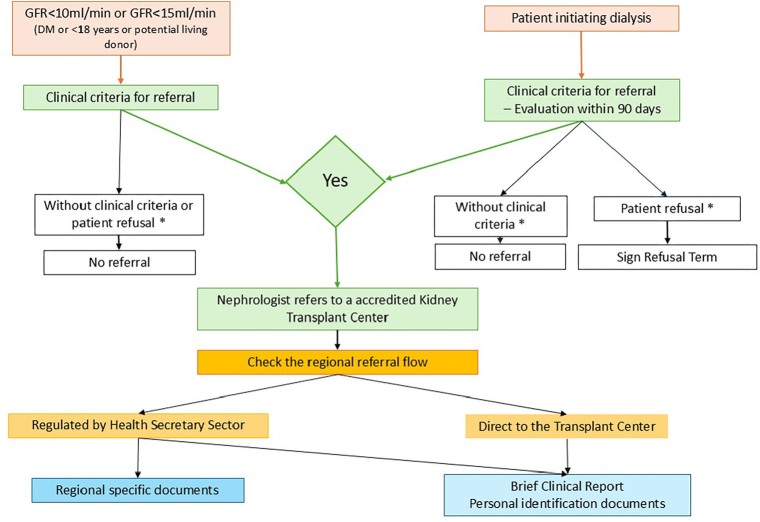
Referral flow for kidney transplant evaluation.

For referral, personal identification documents and a brief clinical report completed by the nephrologist are required^31^. In cities with regional regulation, additional documents may be required, as determined by local authorities. A common practice is to assign a member of the dialysis care team the responsibility for managing referrals and updating the list. Figure 1 provides a schematic overview of the referral flow for kidney transplantation.

Note – *Reassess when there is a change in the patient’s clinical condition or preference.

## Management of Patients on the Kidney Transplant Waiting list

***Provide continuous education on the relevance of kidney transplantation, addressing the steps of the process and the importance of maintaining an optimal clinical, vaccinal, and metabolic status.***

Education strategies are key to expanding access to transplantation^40^. Lack of adequate information about the process and outcomes of kidney transplantation contributes to low enlisting of individuals with CKD for transplantation, as well as inactivation or removal of patients from the waiting list^41^. In addition to unavoidable clinical causes, there are preventable reasons for temporary or permanent removal from the waiting list, such as withdrawal or delay in performing the periodic procedures necessary for remaining on the list. These include medical evaluations, regular complementary tests, and periodic serum collection for immunological screening and panel reactive antibody (PRA) testing^42^.

Thus, it is essential that the multidisciplinary care team continuously promotes health education to minimize socioeconomic disparities that impact health literacy among individuals with CKD, thereby expanding access to transplantation^43,44^.

Chart 1 summarizes the main aspects to be addressed by the multidisciplinary and multi-professional team during the continuous education process of a patient eligible for transplantation.

**Chart 1:** Main topics to be addressed in the continuous education process for kidney transplant candidates^40,41, 42, 43, 44, 45, 46, 47^

Explain the differences between renal replacement therapies, informing patients about the benefits and challenges of kidney transplantation compared to dialysis.
Provide guidance on the need to attend appointments regularly, both for health monitoring and to remain active on the waiting list.
Reinforce the importance of adherence to medications, testing, and guidelines to maintain transplant eligibility.
Inform about the need for proper metabolic and clinical control.
Provide guidance on the importance of adhering to the required vaccinations, in accordance with the National Immunization Program.
Address emotional issues related to transplantation, such as anxiety, as well as the importance of family and social support for better adaptation.
Ensure that contact details, including phone numbers and addresses, are always up to date to facilitate communication with the transplant team.

***Maintain effective communication with the transplant team by reporting episodes that may impact the patient’s transplantability, such as blood transfusions, pregnancies, infections, vaccinations, and changes in clinical status.***

Effective communication between dialysis clinics and transplant centers is crucial to keeping CKD patients active on the transplant waiting list^48^. Certain clinical events may require temporary inactivation or even removal from the list. Therefore, any relevant clinical and surgical events, such as acute myocardial infarction, stroke, ***anticoagulation,*** and neoplasms, should be reported to the transplant center. Events like pregnancy, transfusions, infections, and vaccinations have the potential to cause sensitization and, consequently, modify the PRA, impacting the transplantability of individuals with CKD^49^.

***Ensure quarterly submission of serum samples from patients who are active on the list to the Transplant Immunology Laboratory.***

According to current Brazilian legislation, maintaining a patient as “active” on the waiting list requires the patient to have updated serum samples (collected less than 90 days prior and with no immunizing procedures) and a PRA assessment performed less than 120 days prior^50^. Thus, ensuring the proper collection and timely submission of serum samples for evaluation at the reference histocompatibility laboratory is critical to guaranteeing access to transplantation.

***In the management of anemia, blood transfusions should be reserved for situations of clinical instability, such as tissue hypoxemia, symptomatic anemia, or significant active bleeding.***

Kidney transplantation requires a negative crossmatch, i.e., the absence of a significant reaction of antibodies targeting donor-specific human leukocyte antigens (HLA). The formation of anti-HLA donor-specific antibodies requires prior exposure to HLA molecules, with the major sensitization events being previous transplants, blood transfusions, and pregnancies^51,52^. The presence of antibodies against HLA antigens reduces transplantability and may increase immunological risk and reduce graft survival^53^. Therefore, blood transfusions should be carefully evaluated and reserved for situations of clinical instability in individuals listed for kidney transplantation. There is no robust evidence supporting the use of leukoreduction (leukocyte-filtered concentrates) as a strategy to reduce allosensitization in kidney transplant candidates^54^.

## Dialysis Access Care and Prioritization

***As a criterion for prioritization for kidney transplantation, failure or impossibility of performing peritoneal dialysis must be confirmed, as well as imminent failure of vascular access for hemodialysis.***

***When assessing hemodialysis patients, the vascular access used for hemodialysis and the vascular access required for renal graft implantation should be considered as distinct situations.***

***Refer the patient for prioritization assessment for kidney transplant before exhausting all vascular accesses for hemodialysis.***

***Due to the risk of compromising the venous vascular bed required for kidney graft implantation, prioritization assessment should be considered before using vascular access in the lower limbs (femoral access).***

Despite variations across some Brazilian states, according to regulations set by state transplant centers^55^, the main indication for prioritization for kidney transplantation is the inability to obtain access for dialysis^31^. Current Brazilian legislation establishes as an urgent criterion for kidney transplantation the imminent total and permanent technical impossibility of obtaining access to perform any modality of dialysis. This text is derived from Ordinance No. 2,600, published in 2009^50^, and reaffirmed in the 2017 Technical Standard^31^. It should be clarified, however, that the term “prioritization”, often used in the international literature to refer to placing the patient at the top of the list, refers in this context to the urgency of performing the transplant. Given that, among chronic and permanent organic dysfunctions, CKD is the only condition for which there are effective function replacement therapy options, it is agreed that only the technical impossibility of performing one of the renal function replacement modalities is considered an urgency for prioritization, even though, in some states, other prioritization criteria may apply.

There is a specific situation in the Brazilian context that must be accounted, considering the low utilization rate of peritoneal dialysis as a renal replacement therapy. According to the 2023 Survey of the Brazilian Society of Nephrology, the utilization rate of peritoneal dialysis is around 4%, and in some Brazilian states, the therapy is not even available^1^. For this reason, this working group included among its recommendations the impossibility of performing peritoneal dialysis, encompassing not only clinical contraindications - which are few^56,57^ - or the failure of the technique in patients previously submitted to this modality^58^, but also the local unavailability of the therapy within healthcare systems.

Regarding vascular access, there is little evidence on when access failure should be considered for performing hemodialysis. It is noteworthy that Brazilian legislation considers the imminence of access impossibility as an urgency criterion, and therefore, one should not wait for all possibilities to be exhausted before evaluating the patient for prioritization. Although there is little data in the literature, the consensus of the European Society for Vascular Surgery recommends classifying vascular failure into three categories: **Class I**, in which venous access options in the upper limbs, such as axillary, subclavian, brachiocephalic veins, and superior vena cava, are exhausted; **Class II**, when all options in the upper and lower limbs have been exhausted, including iliac veins and inferior vena cava; and **Class III**, when unconventional access is required, such as translumbar and transhepatic routes^59^.

Vascular access failure for hemodialysis is a serious clinical condition that significantly affects the prognosis of CKD patients. Although transplant outcomes in this group of patients are poorer, kidney transplantation offers higher survival rates compared to other last-resort vascular access alternatives, such as translumbar catheter, femoral vein access, or saphenous vein loops, among others^60,61^. Despite the availability of these alternative resources - as well as prostheses and stents for correcting vascular access inadequacies - it should be considered that kidney transplantation requires the patency of a minimum venous bed for graft implantation. Therefore, by consensus, this working group chose to recommend a distinction between vascular access for hemodialysis and vascular access for renal graft implantation, considering the risk of chronic complications that may preclude the use of femoral veins or the inferior vena cava for performing either orthotopic or even heterotopic kidney transplantation. For this reason, there was an agreement among the working group members that patients should be evaluated by the transplant team for prioritization before the use of vascular accesses in the lower limbs.

## Final Messages

This consensus highlights the importance of constantly improving public policies and health education strategies to expand access to kidney transplantation in Brazil, enabling more patients to benefit from this therapeutic modality. Kidney transplantation should be considered a therapeutic option for patients with advanced CKD whenever there are no clinical contraindications. However, the shortage of donors represents a challenge and demands the optimization of listing and referral processes. Patient selection should consider the expected benefits and risks, as well as the identification of individual and social factors that warrant attention, as these may impact treatment adherence and long-term transplant outcomes.

The benefit of transplantation at the individual level is variable and should be discussed on a case-by-case basis, and organ shortage limits the availability of transplants for all potential candidates. The long-term outcome of kidney transplantation depends, among other factors, on minimizing perioperative complications, carefully balancing the risks and benefits of immunosuppression, and maintaining good adherence to immunosuppressive treatment.

Finally, it is recommended that screening and referral for transplantation should not be delayed, to allow adequate planning and increase the chances of performing a preemptive transplant. The integration of dialysis and transplant services, alongside improved regulation of institutional flows, will be crucial for increasing kidney transplant rates in the country and improving clinical outcomes for the CKD population in Brazil.

The information compiled in this document is expected to stimulate the offer of kidney transplantation as RRT to CKD patients and facilitate the early and effective referral of potential candidates to kidney transplant centers.

## Data Availability

The entire dataset supporting the results of this study was published in the article itself.
