# Supplementary material for: Referral for kidney transplantation: a consensus statement from the Brazilian Society of Nephrology
Source: J Bras Nefrol. 2025 Aug 18;47(4):e20250110. doi: 10.1590/2175-8239-JBN-2025-0110en (PMC12448117; doi:10.1590/2175-8239-JBN-2025-0110en)
Supplement: Translation

# Translation: Encaminhamento para transplante renal: consenso da Sociedade Brasileira de Nefrologia

**DOI:** 10.1590/2175-8239-JBN-2025-0110pt

**Authors:** Helady Sanders-Pinheiro, Luiz Gustavo Modelli de Andrade, Tainá Veras de Sandes-Freitas, Laila Almeida Viana, Lucio Requiao-Moura, Luiz Roberto de Sousa Ulisses, Pedro Tulio Monteiro de Castro de Abreu Rocha, Gustavo Fernandes Ferreira, Lilian Pires do Carmo, Lauro Vasconcelos, Alvaro Pacheco-Silva, José Andrade Moura-Neto

**Affiliations:** 1 Sociedade Brasileira de Nefrologia, São Paulo, SP, Brasil.; 2 Universidade Federal de Juiz de Fora, Faculdade de Medicina, Departamento de Clínica Médica, Juiz de Fora, MG, Brasil.; 3 Serviço de Transplante Renal do Hospital Universitário da Universidade Federal de Juiz de Fora, Juiz de Fora, MG, Brasil.; 4 Universidade Estadual Paulista, Departamento de Medicina Interna, Botucatu, SP, Brasil.; 5 Universidade Federal do Ceará, Faculdade de Medicina, Departamento de Clínica Médica, Fortaleza, CE, Brasil.; 6 Rede D’Or, Hospital São Luiz Itaim, São Paulo, SP, Brasil.; 7 Fundação Oswaldo Ramos, Hospital do Rim, São Paulo, SP, Brasil.; 8 Universidade Federal de São Paulo, Departamento de Medicina, São Paulo, SP, Brasil.; 9 NEFROCLÍNICAS, Brasília, DF, Brasil.; 10 Rede Américas, Hospital São Lucas Copacabana, Rio de Janeiro, RJ, Brasil.; 11 Universidade Federal do Rio de Janeiro, Hospital Universitário Clementino Fraga Filho, Unidade de Terapia Intensiva, Rio de Janeiro, RJ, Brasil.; 12 Santa Casa de Juiz de Fora, Unidade de Transplante, Juiz de Fora, MG, Brasil.; 13 Universidade Federal de Minas Gerais, Faculdade de Medicina, Departamento de Clínica Médica, Belo Horizonte, MG, Brasil.; 14 Hospital Evangélico de Belo Horizonte, Serviço de Transplante Renal, Belo Horizonte, MG, Brasil.; 15 Universidade Federal do Espírito Santo, Faculdade de Medicina, Departamento de Clínica Médica, Vitória, ES, Brasil.; 16 Escola Bahiana de Medicina e Saúde Pública, Departamento de Clínica Médica, Salvador, BA, Brasil.

**Keywords:** Transplante Renal, Doença Renal Crônica, Encaminhamento, Acesso Efetivo aos Serviços de Saúde, Consenso, Tomada de Decisão Clínica

## Abstract

O Brasil está entre os cinco países que mais realizam transplantes renais, operando o maior sistema público de transplantes do mundo. Entretanto, o número de procedimentos realizados representa menos de 40% da demanda, e há grandes disparidades regionais, com áreas do país com atividade transplantadora muito baixa. Entre as potenciais causas para esse cenário estariam a baixa frequência de encaminhamento para avaliação para transplante ou o encaminhamento em condições clínicas inadequadas. A Sociedade Brasileira de Nefrologia propõe, neste documento, reunir as informações que fundamentam a escolha do transplante renal como terapia renal substitutiva, além de detalhar as contraindicações e os cuidados com o paciente após listagem e priorização, alinhados à realidade nacional. Nosso objetivo é fornecer um documento que sirva como referência nacional e contribua para agilizar o encaminhamento ao transplante renal, tornando-o mais frequente e eficaz, ampliando, assim, o acesso dos candidatos a esse tratamento.

## Considerações Iniciais

A doença renal crônica (DRC) avançada representa um desafio crescente para os sistemas de saúde em todo o mundo, incluindo o Brasil. Estima-se que, em 2023, cerca de 157 mil pacientes estavam em terapia dialítica no país^1^, e aproximadamente 33 mil aguardavam, em lista, por um transplante de rim^2^. O transplante renal, quando comparado a outras modalidades de terapia renal substitutiva (TRS), oferece vantagens em termos de sobrevida, qualidade de vida e custo-efetividade^3,4^. No entanto, apesar dos benefícios, o acesso ao transplante no Brasil ainda é limitado por diversos fatores, como as taxas de doação aquém da necessidade, a falta de conscientização sobre a importância da doação, a necessidade de discussão prévia com familiares sobre o desejo individual de doar órgãos^5^, a localização e distribuição dos centros transplantadores, os critérios de seleção e o encaminhamento inadequado dos pacientes.

As disparidades no acesso ao transplante renal e as barreiras que impedem a inscrição de pacientes elegíveis representam desafios para tornar o processo mais eficiente, equitativo e justo. Nesse contexto, o *Encaminhamento Para o Transplante Renal: Consenso da Sociedade Brasileira de Nefrologia* propõe recomendações baseadas em evidências e na opinião de especialistas para orientar a seleção, a identificação de contraindicações, a priorização e o encaminhamento de pacientes, de modo que as decisões sejam fundamentadas em critérios clínicos bem definidos e alinhadas à realidade e à legislação nacionais. Desse modo, oferece-se uma fonte simplificada, acessível e prática para esclarecer as dúvidas dos nefrologistas não transplantadores - profissionais que desempenham um papel estratégico ao iniciar o processo que culmina no acesso do paciente ao transplante renal.

## Transplante Renal Como Terapia Renal Substitutiva

***O transplante renal deve ser apresentado como TRS, assim como as modalidades de diálise, aos pacientes com DRC e risco de progressão, como parte do tratamento conservador, a partir do estágio G4 [taxa de filtração glomerular (TFG) 29-15 ml/min/1,73m^2^].***

Com a progressão da DRC para seu estágio avançado (estágio 5: TFG <15ml/min/1,73m^2^), o transplante renal é a TRS que apresenta melhores resultados^3,4^. Por essa razão, quando possível de ser realizado, é considerado a terapia de escolha^3,4,6^. Esses melhores resultados são mensurados por maior sobrevida^4^, melhor qualidade de vida^3,7^, menor morbidade^3,4^ e menores custos^8^. O benefício em termos de aumento de sobrevida tem sido documentado há décadas^9^ e permanece evidente em estudos recentes, que revelam uma sobrevida 55% superior após o transplante renal, em comparação àquela dos pacientes que permanecem em lista de espera – os quais, em geral, são os mais saudáveis entre os que estão em diálise^3,4^. A sobrevida superior é observada mesmo em doadores falecidos classificados como não ótimos ou de critério expandido^10^ (idade >60 anos ou entre 50–59 anos, com história de hipertensão arterial, causa de óbito cerebrovascular ou creatinina no momento da doação >1,5mg/dl)^11,12^. Observou-se uma mudança epidemiológica nos pacientes diagnosticados com DRC e, por conseguinte, nos candidatos a transplante renal, com aumento da proporção de pacientes com maior morbidade. Uma melhor sobrevida também foi observada nesses grupos, incluindo idosos^13,14^, pacientes diabéticos^15^, com múltiplas comorbidades^14^, em diálise por longo período (>10 anos)^16^ ou sensibilizados^17^, e ainda em centros transplantadores de menor atividade^18^. Além da maior sobrevida, o transplante renal está associado a desfechos mais percebidos e valorizados pelos pacientes, como melhor qualidade de vida^3,19^, medida, por exemplo, pela maior participação em atividades comuns da vida^7^ e menor morbidade (e.g., internações)^3,4^. Do ponto de vista dos sistemas de saúde, o transplante renal, em geral, apresenta custos menores e é considerado custo-efetivo, especialmente em casos de maior complexidade, como os realizados com doadores muito limítrofes [*Kidney Donor Profile Index* (KDPI) >85]^8^. Em pacientes pediátricos, o transplante renal também promove melhor sobrevida^20^, além de melhorias na curva de crescimento^21^, no desenvolvimento neurocognitivo^22^ e maior qualidade de vida^23^.

Os estudos que demonstram a superioridade dos resultados do transplante renal em relação a outras TRS, no entanto, basearam-se principalmente em dados de países desenvolvidos com elevados níveis econômicos e sociais^6^. Sabemos, porém, que a mortalidade anual em diálise no Brasil, segundo dados evolutivos do Censo Brasileiro de Diálise, variou de 17,1% a 20,3% nos últimos 8 anos^1^, sendo, portanto, semelhante à descrita nos outros países estudados. Por outro lado, a sobrevida em curto (1 ano) e médio prazo (5 anos) após o transplante renal, segundo dados do último Registro Brasileiro de Transplantes (RBT) da Associação Brasileira de Transplante de Órgãos (ABTO), é de 97% e 93% para enxertos de doadores vivos, e de 92% e 83% para enxertos de doadores falecidos, respectivamente^2^. As taxas de sobrevida brasileiras são, portanto, semelhantes às reportadas por estudos de outros países^24^. Considerando os dados dos dois censos e, diante da inexistência de estudos comparativos brasileiros, podemos hipotetizar que o benefício de maior sobrevida após o transplante renal possa ser extrapolado também para a população brasileira. No cenário nacional, uma análise de dados secundários do estudo multicêntrico transversal ADERE BRASIL, que incluiu 1105 receptores de transplante renal, avaliou a qualidade de vida utilizando o instrumento WHOQOL-BREF. Os resultados mostraram que os receptores brasileiros de transplante renal apresentaram excelente qualidade de vida nos quatro domínios avaliados, com destaque para os domínios físico e das relações sociais^25^. Em consonância, as Diretrizes Clínicas para o Cuidado ao Paciente com DRC no Sistema Único de Saúde, do Ministério da Saúde, recomendam que o transplante renal seja oferecido como TRS^26^.

Em resumo, pode-se considerar que a opção pelo transplante renal como TRS deve ser oferecida a pacientes com DRC com expectativa de progressão para fases avançadas^6^.

## Indicações de Transplante Renal

***Em pacientes com indicação, o encaminhamento deve ser feito o mais precocemente possível, de acordo com as condições clínicas do candidato ao transplante e respeitando a opção do paciente. Dois aspectos devem ser considerados: o momento do encaminhamento para um serviço de transplante e os critérios definidos na legislação brasileira para inscrição em lista.***

***Os potenciais candidatos a transplante renal devem ser encaminhados para um serviço especializado quando a TFG estiver abaixo de 20 ml/min/1,73m^2^.***

***Para a inscrição em lista, os critérios definidos na legislação brasileira são:***

***I – Pacientes que estejam realizando alguma das modalidades de TRS (hemodiálise ou diálise peritoneal); ou***

***II – Pacientes com TFG inferior a 10 ml/min/1,73m^2^; ou***

***III- Pacientes com idade inferior a 18 anos que apresentem TFG menor que 15 mL/min/1,73m^2^; ou***

***IV -Pacientes com diabetes mellitus que apresentem TFG inferior a 15 ml/min/1,73m^2^.***

***No caso de candidatos ao transplante com doador vivo, a avaliação dos doadores pode ser iniciada em pacientes que já estejam em TRS ou que apresentem TFG inferior a 15 ml/min/1,73m^2^.***

Baseado nos potenciais benefícios do transplante renal como TRS, o encaminhamento deve ser precoce. As Diretrizes Clínicas para o Cuidado ao Paciente com Doença Renal Crônica no Sistema Único de Saúde recomendam que os pacientes sejam encaminhados desde o estágio G5, ou seja, com TFG abaixo de 15 ml/min/1,73m^2 6^. Entretanto, não há consenso sobre qual é o momento exato para o encaminhamento do paciente a um serviço especializado em transplante renal. Idealmente, os potenciais candidatos a transplante renal devem ser encaminhados para avaliação pelo menos seis a doze meses antes do início previsto da diálise, a fim de facilitar a identificação/investigação de doadores vivos e planejar um possível transplante preemptivo^6^. O objetivo é reduzir o risco de óbito precoce, possibilitando o acesso oportuno ao transplante renal. Apesar da ausência de consenso, considerando os benefícios do transplante preemptivo, este grupo de trabalho recomenda que os pacientes sejam encaminhados com TFG abaixo de 20 ml/min/1,73m^2^.

O transplante preemptivo oferece melhores desfechos clínicos após o procedimento, melhor qualidade de vida e benefícios econômicos quando comparado à diálise. Está associado a uma maior sobrevida do enxerto e menor incidência de rejeição aguda, permitindo também que os pacientes sejam poupados dos riscos potenciais associados à terapia de diálise, como infecção relacionada ao cateter, efeitos adversos cardiovasculares e complicações intradialíticas, como hipotensão^27,28^. Apesar dos benefícios, o transplante preemptivo não é amplamente realizado ao redor do mundo. No período de 2000 a 2018, nos Estados Unidos, menos de 10% (9,3%) dos transplantes renais foram realizados de forma preemptiva. Relatos de outros países revelam taxas ainda mais baixas: Espanha (5%), Uruguai (5,4%) e Indonésia (2,7%)^28^. O Brasil não possui registros precisos quanto ao número de transplantes preemptivos realizados; no entanto, dados do estudo ADERE BRASIL evidenciaram que apenas 3,3% dos 1105 pacientes incluídos foram transplantados de forma preemptiva^29^. Algumas razões podem explicar o número reduzido para essa modalidade de transplante: políticas de alocação de órgãos, educação do paciente e da equipe de atendimento, e encaminhamento tardio ao nefrologista^30^. No Brasil, o transplante renal preemptivo pode ser realizado não apenas com doadores vivos, mas também com doadores falecidos, em pacientes que apresentem TFG <10 ml/min/1,73m^2^, ou < 15 ml/min/1,73m^2^ no caso de menores de 18 anos e pacientes diabéticos, conforme citado anteriormente^31^.

No serviço de transplante, após avaliação clínica e laboratorial, os potenciais candidatos podem ser inscritos na lista de transplante, de acordo com os critérios definidos pela legislação vigente^31^. Pacientes com diabetes mellitus e crianças representam situações especiais, pois a morbimortalidade aumenta de forma considerável à medida que a TFG diminui. Por essa razão, preconiza-se a inscrição em lista com TFG <15ml/min/1,73m^2 6,31^. Após o início da TRS, os pacientes com menor tempo em diálise evoluem com menor risco de óbito e de perda do enxerto após o transplante renal^32, 33, 34^. Essa diferença já é observada mesmo com tempos relativamente curtos, como 6 meses de tratamento dialítico, e aumenta progressivamente a cada ano adicional de terapia. O risco de óbito aumenta de 18 a 68% entre pacientes com 6 meses a >5 anos em diálise, respectivamente, enquanto o risco de perda do enxerto varia de 25 a 46% nesses mesmos períodos^32^. As Diretrizes Clínicas para o Cuidado ao Paciente Com Doença Renal Crônica – DRC no Sistema Único De Saúde preconizam o prazo de até 90 dias após o início da diálise para notificação oficial sobre a opção pelo transplante renal, como forma de agilizar e garantir o acesso ao encaminhamento. Essa opção poderá ser alterada a qualquer momento, pelo paciente ou em decorrência de sua condição clínica^26,35^.

O transplante simultâneo pâncreas-rim pode ser considerado para pacientes com diabetes mellitus insulino-dependente (tipo I, diabetes autoimune latente do adulto, tipo MODY ou quadro de início precoce) combinado à DRC em diálise ou com taxa de filtração glomerular inferior a 20ml/min/1,73m^2^. Quando há a necessidade de transplante combinado de rim com qualquer outro órgão (fígado e coração), a alocação dos enxertos segue a legislação específica do outro órgão^31^.

## Contraindicações: Absolutas, Relativas e Temporárias

***Podem ser consideradas contraindicações absolutas que, portanto, não justificam encaminhamento para avaliação para transplante renal: neoplasias malignas não tratadas, doença neurológica degenerativa progressiva, vasculopatia grave envolvendo as artérias ilíacas, doença psiquiátrica mal controlada, uso abusivo de substâncias ilícitas, insuficiências orgânicas sem condição de tratamento ou com indicação de transplante combinado (coração e fígado).***

***Podem ser consideradas contraindicações relativas ou temporárias: doença arterial coronariana não tratada; perda por causa imunológica recente (< 6 meses); acidente vascular encefálico (AVE), infarto agudo do miocárdio (IAM) ou ataque isquêmico transitório (AIT) recentes (< 6 meses para AVE/IAM; < 3 meses para AIT); atividade extra-renal de doença sistêmica; doença ulcerosa péptica em atividade; infecção aguda; anticoagulação plena que não possa ser suspensa ou revertida; e dupla antiagregação plaquetária.***

***No caso das situações classificadas como temporárias ou relativas, sugere-se aguardar a resolução/evolução do quadro antes do encaminhamento.***

***Dada a complexidade e a dificuldade de generalização, recomenda-se sugerir ao paciente que busque uma segunda opinião, caso seja considerado contraindicado para transplante renal após avaliação.***

***Como a decisão é individualizada e baseada na avaliação de uma equipe multidisciplinar, em caso de dúvidas, sugerimos encaminhar o paciente, para o que serviço de transplante possa deliberar. Alternativamente, pode-se entrar em contato com o serviço e buscar orientação.***

A consideração de que um paciente não possui condições clínicas para ser avaliado para transplante renal é um tema que gera considerável discussão, uma vez que os critérios e limites para essa avaliação não são claramente definidos^6^. Para fins deste consenso, dividiremos as contraindicações para encaminhamento em: **1. Absolutas**: aquelas em que praticamente não há dúvidas quanto à ausência de benefício ou em que o risco associado ao transplante renal é muito elevado; **2. Relativas**: o benefício é provável, mas o risco também é elevado, embora contornável; nesses casos, o aceite da candidatura varia entre os centros transplantadores; **3. Temporárias**: condições de risco elevado, porém com resolução provável em curto ou médio prazo. A maioria dos centros não aceitará como candidatos os pacientes com contraindicações absolutas, portanto, sugere-se que não sejam encaminhados. No caso das situações classificadas como temporárias ou relativas, recomenda-se aguardar a resolução/evolução do quadro antes do encaminhamento^6^.

Há algumas situações que tornam o transplante mais desafiador, eventualmente até reduzindo sua taxa de sucesso, como o transplante em indivíduos idosos^36^, obesos^37^, pessoas vivendo com infecção pelo vírus da imunodeficiência adquirida (HIV)^38^, hepatite B, hepatite C^39^ ou na presença de múltiplas comorbidades^14^. Entretanto, ao se avaliar o benefício do transplante renal, o comparador não deve ser um transplante realizado em cenário ideal, mas sim a permanência em diálise. Nesse sentido, as evidências disponíveis até o momento têm demonstrado, de forma consistente, que o transplante beneficia a maior parte desses pacientes, os quais devem, portanto, ser encaminhados para avaliação pré-transplante^14,36, 37, 38, 39^. Durante tal avaliação, a equipe realiza uma abordagem integral do paciente e, sendo ele considerado elegível, segue com as orientações para controle ou mitigação das condições passíveis de manejo, como comorbidades e doenças virais.

Quanto às neoplasias, se o paciente estiver com um câncer em atividade, sem perspectiva de tratamento, ou em tratamento quimioterápico ou radioterápico vigente, este não deve ser encaminhado para transplante neste momento. Entretanto, após a finalização do tratamento, a indicação do transplante dependerá do tipo de câncer e do tempo decorrido desde o término do tratamento. Dada a complexidade dessa avaliação, sugerimos que o paciente seja encaminhado ao centro de transplante, onde uma equipe multidisciplinar avaliará o risco-benefício individualmente^6^.

## Fluxo de Encaminhamento

***Pacientes que atendam aos critérios clínicos para o transplante devem ser encaminhados aos centros habilitados, seguindo a recomendação local, onde serão submetidos*** à ***avaliação para posterior inclusão em lista.***

Para o encaminhamento, são necessários documentos de identificação pessoal e relatório clínico sucinto, preenchido pelo nefrologista^31^. Em cidades com regulação regional, podem ser exigidos documentos adicionais, definidos a critério da gestão local. Uma prática bastante comum é delegar a um membro da equipe de cuidado do paciente em diálise a responsabilidade pela gestão do encaminhamento e pela manutenção na lista. A Figura 1 apresenta um resumo esquemático do fluxo de encaminhamento para transplante renal.

**Figura 1:** Fluxo de encaminhamento para avaliação para transplante renal

Nota – *Reavaliar quando houver mudança nas condições clínicas ou na opção do paciente.

## Manejo do Paciente em Lista Para Transplante Renal

***Realizar educação continuada sobre a relevância do transplante renal, abordando as etapas do processo e a importância de manter um estado clínico, vacinal e metabólico otimizados.***

Estratégias de educação são fundamentais para ampliar o acesso ao transplante^40^. A falta de informação adequada sobre o processo e os resultados do transplante renal tem impacto na baixa listagem de indivíduos com DRC para transplante, bem como na inativação ou remoção de pacientes da lista de espera^41^. Além das causas clínicas inevitáveis, existem motivos evitáveis para a saída temporária ou definitiva da lista de espera, como a desistência ou o retardo na realização dos procedimentos periódicos necessários para manutenção em lista, incluindo avaliação médica, exames complementares regulares e coleta periódica de soro para seleção imunológica e painel de anticorpos reativos (PRA)^42^.

Assim, é fundamental que a equipe multiprofissional assistente atue continuamente na promoção da educação em saúde, a fim de minimizar as disparidades socioeconômicas que impactam no letramento em saúde dos indivíduos com DRC e, dessa forma, ampliar o acesso ao transplante^43,44^.

O Quadro 1 resume os principais aspectos a serem abordados pela equipe multidisciplinar e multiprofissional durante o processo de educação continuada de um paciente candidato ao transplante.

**Quadro 1:** Principais tópicos a serem abordados no processo de educação continuada de candidatos a transplante renal^40,42, 43, 44, 45, 46, 47^

Explicar as diferenças entre as terapias renais substitutivas, informando benefícios e desafios do transplante renal em comparação com a diálise.
Orientar sobre a necessidade de comparecimento regular às consultas, tanto para o monitoramento da saúde quanto para a manutenção na lista.
Reforçar a importância da aderência às medicações, exames e orientações, a fim de manter a elegibilidade ao transplante.
Informar sobre a necessidade de controle metabólico e clínico adequado.
Orientar quanto à importância de manter aderência às vacinas requeridas, de acordo com o Programa Nacional de Imunizações.
Abordar questões emocionais relacionadas ao transplante, como ansiedade, bem como a importância do suporte familiar e social para uma melhor adaptação.
Garantir que os dados de contato, incluindo telefones e endereços, estejam sempre atualizados, para facilitar a comunicação com a equipe de transplante.

***Manter comunicação eficaz com a equipe de transplante, informando episódios que possam impactar a transplantabilidade do paciente, como transfusões sanguíneas, gestações, infecções, vacina*** çõe ***s e mudanças do status clínico.***

A comunicação eficaz entre clínicas de diálise e centros de transplante é crucial para manter pacientes com DRC ativos na lista de espera para transplante^48^. Determinados eventos clínicos podem requerer inativação temporária ou mesmo remoção da lista. Por isso, devem ser comunicados ao centro de transplante quaisquer eventos clínicos e cirúrgicos relevantes, como infarto agudo do miocárdio, acidente vascular cerebral, anticoagulação e neoplasias. A ocorrência de gestações, transfusões, infecções e vacinas tem potencial de sensibilização e, portanto, de modificação do PRA, impactando a transplantabilidade do indivíduo com DRC^49^.

***Garantir o envio trimestral do soro do paciente ativo em lista para o Laboratório de Imunologia de Transplante.***

De acordo com a legislação brasileira vigente, a manutenção do paciente “ativo” na lista de espera requer que o paciente tenha soro atualizado (com menos de 90 dias de coleta e ausência de procedimento imunizante) e avaliação do PRA com menos de 120 dias^50^. Assim, garantir a coleta e o envio adequados do soro para avaliação no laboratório de histocompatibilidade de referência é fundamental para assegurar o acesso ao transplante.

***No manejo da anemia, recomenda-se reservar a transfusão sanguínea para situações de instabilidade clínica, como hipoxemia tecidual, anemia sintomática ou sangramento ativo significativo.***

A realização do transplante renal requer uma prova cruzada negativa, ou seja, a ausência de reação significativa de anticorpos contra os antígenos leucocitários humanos (HLA) específicos do doador. A formação de anticorpos anti-HLA contra o doador requer exposição prévia a moléculas HLA, sendo as principais formas de sensibilização os transplantes prévios, transfusões sanguíneas e gestações^51,52^. A presença de anticorpos contra os antígenos HLA reduz a transplantabilidade, além de poder aumentar seu risco imunológico e reduzir a sobrevida do enxerto^53^. Assim, as transfusões sanguíneas devem ser criteriosamente avaliadas e reservadas para situações de instabilidade clínica em indivíduos listados para o transplante renal. Não há evidências robustas que suportem o uso da leucorredução (concentrados com filtro de leucócitos) como estratégia para reduzir a alossensibilização em candidatos ao transplante renal^54^.

## Cuidados com Acesso Para Diálise e Priorização

***Como critério de priorização para o transplante renal, deve-se constatar falência ou a impossibilidade para realização da modalidade de diálise peritoneal, bem como a iminência de falência do acesso vascular para realização da hemodiálise.***

***Na avaliação do paciente em hemodiálise, devem-se considerar como situações distintas o acesso vascular utilizado para a hemodiálise e o acesso vascular necessário para o implante do enxerto renal.***

***Encaminhar o paciente para avaliação de priorização para o transplante renal antes do esgotamento de todos os acessos vasculares para realização de hemodiálise.***

***Devido ao risco de comprometimento do leito vascular venoso necessário para o implante do enxerto renal, deve-se considerar a avaliação de priorização antes do uso de acessos vasculares em membros inferiores (acessos femorais).***

Apesar de variações em alguns estados brasileiros, de acordo com as normas das centrais estaduais de transplante^55^, a principal indicação de priorização para o transplante renal é a impossibilidade de acesso para a realização de diálise^31^. A legislação brasileira vigente estabelece como critério de urgência para a realização de transplante renal a iminência de impossibilidade técnica total e permanente para a obtenção de acesso para qualquer das modalidades de diálise. Tal texto é derivado da Portaria 2.600, publicada em 2009^50^, e reafirmado na Norma Técnica de 2017^31^. Deve-se esclarecer, entretanto, que o termo *priorização*, frequentemente utilizado na literatura internacional para se referir ao posicionamento do paciente no topo da lista, diz respeito, neste contexto, à urgência para a realização do transplante. Considerando que, dentre as disfunções orgânicas crônicas e permanentes, a DRC é a única que apresenta opções eficazes de tratamento de substituição de função, de consenso, apenas a impossibilidade técnica para a realização de uma das modalidades de substituição de função renal é considerada uma urgência para priorização, mesmo que, em alguns estados, outros critérios de priorização sejam aplicados.

Há uma situação específica no cenário brasileiro que deve ser considerada, tendo em vista a baixa taxa de utilização da diálise peritoneal como TRS. De acordo com o Censo da Sociedade Brasileira de Nefrologia de 2023, a taxa de utilização da diálise peritoneal está em torno de 4%, sendo que em alguns estados brasileiros a terapia nem sequer está disponível^1^. Por esse motivo, este grupo de trabalho incluiu, entre suas recomendações, a impossibilidade de realização da diálise peritoneal, abrangendo não apenas as contraindicações clínicas - que são poucas^56,57^ - ou a falência da técnica em pacientes previamente submetidos a essa modalidade^58^, mas também a indisponibilidade local da terapia por parte dos sistemas de saúde.

No que diz respeito ao acesso vascular, há poucas evidências sobre quando se deve considerar a falência para a realização da hemodiálise. Destaca-se que a legislação brasileira considera como critério de urgência a iminência de impossibilidade de acesso, e, portanto, não se deve aguardar o esgotamento de todas as possibilidades para que o paciente seja avaliado para a priorização. Apesar de existirem poucos dados na literatura, o consenso da Sociedade Europeia de Cirurgia Vascular recomenda a classificação da falência vascular em três classes: a **classe I**, em que há esgotamento das opções de acesso venoso nos membros superiores, tais como veias axilares, subclávias, braquiocefálicas e veia cava superior; a **classe II**, em que se esgotam as opções nos membros superiores e inferiores, incluindo veias ilíacas e veia cava inferior; e a **classe III**, quando há a necessidade de acesso não convencional, como os translombares e transhepáticos^59^.

A falência de acesso vascular para hemodiálise é uma condição clínica grave que impacta significativamente o prognóstico de pacientes com DRC. Embora os resultados do transplante nesse grupo de pacientes sejam inferiores, a realização de um transplante renal apresenta sobrevidas superiores em comparação às demais alternativas de acesso vascular de última linha, como o cateter translombar, veia femoral, alça de safena, entre outros^60,61^. Apesar da disponibilidade desses recursos alternativos, bem como de próteses e *stents* para correções de inadequações em acessos vasculares, deve-se considerar que a realização do transplante renal pressupõe a patência de uma rede venosa mínima para o implante do enxerto. Sendo assim, por consenso, este grupo de trabalho optou por recomendar uma distinção entre o acesso vascular para a realização de hemodiálise e o acesso vascular para o implante do enxerto renal, considerando o risco de complicações crônicas que possam inviabilizar o uso de veias femorais ou da veia cava inferior para a realização do transplante renal ortotópico ou mesmo heterotópico. Por esse motivo, houve entendimento comum entre os membros do grupo de trabalho de que os pacientes devem ser avaliados pela equipe de transplante com vistas à priorização antes da utilização de acessos vasculares em membros inferiores.

## Mensagens Finais

Este consenso destaca a importância do aprimoramento constante das políticas públicas e das estratégias de educação em saúde para ampliar o acesso ao transplante renal no Brasil, permitindo que mais pacientes possam se beneficiar dessa modalidade terapêutica. O transplante renal deve ser considerado uma opção terapêutica para pacientes com DRC avançada sempre que não houver contraindicação clínica. A escassez de doadores, entretanto, representa um desafio e exige a otimização dos processos de listagem e encaminhamento. A seleção dos pacientes deve considerar os benefícios e riscos esperados, bem como a identificação de fatores individuais e sociais que merecem atenção, pois podem impactar a aderência ao tratamento e os resultados do transplante em longo prazo.

O benefício do transplante em nível individual é variável e deve ser discutido caso a caso, e a escassez de órgãos limita a disponibilidade de um transplante para todos os potenciais candidatos. O resultado do transplante renal em longo prazo depende, dentre outros fatores, da minimização de complicações perioperatórias, do balanço cuidadoso entre riscos e benefícios da imunossupressão e da manutenção de uma boa aderência ao tratamento imunossupressor.

Por fim, recomenda-se que a triagem e o encaminhamento para o transplante não ocorram de forma tardia, de modo a permitir um planejamento adequado e aumentar as chances de realização de um transplante preemptivo. A integração entre os serviços de diálise e transplante, aliada a uma melhor regulamentação dos fluxos institucionais, será determinante para a ampliação das taxas de transplante renal no país e para a melhoria dos desfechos clínicos da população com DRC no Brasil.

Espera-se que as informações reunidas neste documento possam estimular a oferta do transplante renal como TRS aos pacientes com DRC e facilitar o encaminhamento precoce e efetivo dos potenciais candidatos aos centros de transplante renal.

## Data Availability

Todo o conjunto de dados que dá suporte aos resultados deste estudo foi publicado no próprio artigo.
